# Nivolumab plus ipilimumab in the treatment of advanced melanoma

**DOI:** 10.1186/s13045-015-0219-0

**Published:** 2015-10-31

**Authors:** Katy K. Tsai, Adil I. Daud

**Affiliations:** Helen Diller Comprehensive Cancer Center, University of California, 1600 Divisadero St., Box 1770, San Francisco, CA 94115 USA

**Keywords:** Melanoma, Immunotherapy, PD-1, CTLA-4, Nivolumab, Ipilimumab

## Abstract

Advanced melanoma has historically been a difficult disease to treat due to few effective systemic treatment options. However, over the past few years, scientific advancements in immune checkpoint inhibition have resulted in several novel approaches that have changed front-line management of advanced melanoma. Despite these exciting developments, there remains room for improvement in treatment outcomes. Combination immunotherapy, in particular combined cytotoxic T-lymphocyte-associated protein 4 (CTLA-4) and programmed death 1 (PD-1) blockade, represents an important first step in this direction.

## Background

Advanced melanoma has historically been a difficult disease to treat, owing to few systemic treatment options. Over the past few years, however, scientific advancements have resulted in the approval of novel therapeutic approaches that have changed front-line management of advanced melanoma, with immune checkpoint inhibition generating arguably the most excitement in the field of melanoma oncology and beyond. Pioneering work by James Allison led to the development of ipilimumab [1, 2], a fully human monoclonal antibody against cytotoxic T-lymphocyte-associated protein 4 (CTLA-4), which was approved by the FDA in 2011 for the treatment of patients with advanced melanoma. Similarly groundbreaking work by Tasuku Honjo resulted in the first description of the programmed death 1 (PD-1) receptor [3], which has since led to the development of two anti-PD-1 antibodies: nivolumab and pembrolizumab. Both antibodies received FDA approval in 2014 for the treatment of advanced melanoma patients following progression after BRAF-targeted therapy (for those with BRAF V600-mutated melanoma) and after ipilimumab. Despite these exciting developments, there remains much work to be done to improve treatment outcomes, and combination immunotherapy seems to hold the key to achieving this goal. CTLA-4 and PD-1 inhibit antitumor immunity via complementary, nonredundant pathways [4], and preclinical models have supported that combined checkpoint blockade synergistically improves antitumor responses compared to blockade of either pathway alone [5–7]. Based on these observations, phase 1 and 2 trials were conducted with combined nivolumab and ipilimumab [8, 9]. Favorable results from these early phase trials led to CheckMate 067, a phase 3 study designed to evaluate the safety and efficacy of nivolumab alone or nivolumab plus ipilimumab in comparison with ipilimumab alone, in patients with previously untreated metastatic melanoma [10]. The recently published landmark results of this trial, as well as its implications for the future of melanoma treatment, will be discussed here.

## Main text

As monotherapies, ipilimumab and nivolumab both clearly show significant activity in the treatment of advanced melanoma. The regulatory approval of ipilimumab was based on phase 3 trial data showing significantly increased median overall survival (OS) in previously treated melanoma patients who received ipilimumab compared to those who received an experimental peptide vaccine alone (10.1 vs 6.4 months; hazard ratio (HR) = 0.66; *p* = 0.003) [11]. Another phase 3 trial, this time conducted in the first-line setting, showed that ipilimumab combined with dacarbazine improved survival compared with dacarbazine alone [12]. With regards to the anti-PD-1 drugs, nivolumab was the first fully human monoclonal antibody against PD-1 to be evaluated therapeutically. In a phase 3 trial, previously untreated patients received nivolumab or dacarbazine, and nivolumab treatment was associated with superior median progression-free survival (PFS) (5.1 vs 2.2 months, *p* < 0.001) and OS at 1 year (72.9 vs 42.1 %, *p* < 0.001) [6]. In another phase 3 trial, patients previously treated with ipilimumab received nivolumab or chemotherapy, with the nivolumab group showing superior overall response rate (ORR) (32 vs 11 %) and fewer grades 3 to 4 adverse events (9 vs 31 %) [13, 14].

While these data show that immunotherapy clearly results in better treatment outcomes compared to chemotherapy, it remains unclear which patients will respond, and the need for predictive models and biomarkers remains [15]. Much research has focused on how to improve the efficacy of currently available immunotherapies. Combined immune checkpoint blockade with anti-CTLA-4 and anti-PD-1 agents represents an important step in this direction, with published phase 1 and phase 2 data indicating the therapeutic promise of this combination. In the phase 1 dose-escalation study of advanced melanoma patients receiving the combination of nivolumab and ipilimumab, the updated ORRs for patients treated with concurrent nivolumab plus ipilimumab were 42 to 43 %. Prolonged duration of response was also seen, with OS rate of 85 % at 1 year and 79 % at 2 years [8, 16]. In the randomized phase 2 study comparing nivolumab plus ipilimumab with ipilimumab alone in patients with *BRAF* wild-type melanoma, ORR was 61 % with combination therapy compared to 11 % with monotherapy, with complete responses seen in 22 and 0 % of patients, respectively [9]. PFS was significantly prolonged with combination therapy (median not reached, compared with 4.4 months in the ipilimumab alone group). Treatment-related adverse events of grade 3 or 4 were 54 % of patients in the combination therapy group, compared with 24 % in the ipilimumab alone group.

To confirm and extend these results, a double-blind, multicenter phase 3 trial (CheckMate 067) was subsequently conducted. In this trial, the safety and efficacy of nivolumab plus ipilimumab with nivolumab and ipilimumab monotherapies were compared, and results were presented at the ASCO 2015 plenary session and published in the *New England Journal of Medicine* [10]. CheckMate 067 enrolled 945 treatment-naïve patients with advanced melanoma who were stratified according to disease stage, BRAF mutation status, and programmed death ligand 1 (PD-L1) expression. Patients underwent 1:1:1 randomization to three different arms: (1) nivolumab 3 mg/kg every 2 weeks with ipilimumab-matched placebo; (2) nivolumab 1 mg/kg every 3 weeks with ipilimumab 3 mg/kg every 3 weeks for four doses, followed by nivolumab 3 mg/kg every 2 weeks; or (3) ipilimumab 3 mg/kg every 3 weeks for four doses with nivolumab-matched placebo. The study was powered to detect differences in PFS and OS for nivolumab plus ipilimumab versus ipilimumab alone and nivolumab alone versus ipilimumab alone; at a median follow-up of 12 months, safety and efficacy data are reported while OS data are at present insufficiently mature. The median PFS for patients taking combination therapy was significantly prolonged at 11.5 months compared to 2.9 months with ipilimumab alone (HR 0.42 (0.31–0.57)) and 6.9 months with nivolumab alone (HR 0.57 (0.43–0.76)). Although the study was not powered to compare combination therapy with nivolumab alone, an exploratory analysis was done which showed the median PFS of the combination to be superior compared to nivolumab alone (HR 0.74 (0.60–0.92)). In addition, combination therapy seems to be superior compared to nivolumab and ipilimumab monotherapies based on ORR: 57.6 % of patients in the combination group, 43.7 % in the nivolumab alone group, and 19 % in the ipilimumab alone group. In addition, though the impressive 80 % tumor regressions with combination therapy observed in the phase 1 study was not reproduced here, there was still significant reduction in tumor burden in the combination, nivolumab alone, and ipilimumab alone groups of −51.9, −34.5, and −5.9 % from baseline, respectively.

PFS data stratified by patient subgroups are also presented; while BRAF mutation status and metastatic burden subgroups all showed benefit from combination or nivolumab monotherapy compared to ipilimumab alone, the data stratified by PD-L1 expression are of particular interest. In the phase 2 trial, a cutoff of ≥ 5 % was used to determine PD-L1 positivity. Results from that trial noted an ORR of 58 % for patients on combination therapy whose tumors were PD-L1-positive, compared with 55 % in those whose tumors were PD-L1-negative. In CheckMate 067, an identical cutoff point for PD-L1 positivity was used though differences in ORR as well as PFS were observed between treatment groups. Patients with PD-L1-positive tumors treated with combination, nivolumab, and ipilimumab had ORRs of 72, 58, and 21 %, respectively (which translated to a PFS benefit as well, with median PFS of 14, 14, and 3.9 months, respectively). For those patients with PD-L1-negative tumors, ORRs were 55, 44, and 18 %, respectively (PFS of 11.2, 5.3, and 2.8 months, respectively).

These improved results with combination therapy; however, this did not come without cost. Although no new safety signals were identified, increased toxicity was seen in the combination group: grades 3–4 adverse events occurred in 55 % of patients, compared to 16.3 % in patients treated with nivolumab alone, and 27.3 % of patients treated with ipilimumab. Treatment-related adverse events leading to therapy discontinuation occurred in 36.4, 7.7, and 14.8 % of patients, respectively (most commonly diarrhea, fatigue, and pruritus).

So, what are some important lessons we can take away from CheckMate 067? Firstly: this data is extremely promising and offers hope for improved treatment for patients with advanced melanoma. However, this data remains preliminary at this point; OS data will hopefully be forthcoming and help illuminate whether combined immune checkpoint blockade in the first-line setting should be the new standard. Assuming for the moment that OS data correlates with the PFS data, the information from this trial could be practice-changing: given the significant improvements in ORR and PFS in the combination and nivolumab alone groups over ipilimumab alone, ipilimumab alone should no longer be used in the front-line setting. An anti-PD-1 agent or, arguably, combined anti-PD-1 and anti-CTLA-4 blockade, should be considered as the first-line therapy.

It is important to note, however, that although the combination of nivolumab and ipilimumab showed improvement in ORR and PFS over nivolumab alone, that the study was not designed specifically to show this difference and more guidance is needed to clarify who will benefit from which upfront therapy. The subgroup analysis with PD-L1 expression does offer some insight into patterns of response. PD-L1-positive patients who received combination or nivolumab alone had the same PFS of 14 months. PD-L1-negative patients who received nivolumab alone had a lower PFS compared to those who received combination therapy (5.3 vs 11.2 months). Given this information, especially in light of the increased toxicity with the combination, it may make sense to tailor the treatment plan based on PD-L1 expression level. For example, PD-L1-negative patients should receive combination therapy to maximize response as long as they are able to tolerate the adverse effects, while PD-L1-positive patients could have an excellent response to nivolumab monotherapy and avoid unnecessary toxicity. PD-1 monotherapy would of course remain a valid option for any patient where excess toxicity could be a concern, regardless of their PD-L1 status. At this time, PD-L1 expression remains an unvalidated biomarker, and research efforts are ongoing to identify biomarkers of response, particularly in the tumor microenvironment.

## Conclusion

In summary, this is an exciting time for melanoma research. Updated data from CheckMate 067, as well as results from other trials that are investigating combination immunotherapy, could move anti-PD-1 agents and combinations thereof into the first-line treatment setting. Longer follow-up and updated overall survival data, as well as more correlative studies, are needed to determine whether such a change is warranted.

## Abbreviations

ASCO: American Society of Clinical Oncology
CTLA-4: cytotoxic T-lymphocyte-associated protein 4
FDA: Food and Drug Administration
HR: hazard ratio
ORR: overall response rate
OS: overall survival
PD-1: programmed death 1
PD-L1: programmed death ligand 1
PFS: progression-free survival
